# Multiple Infectious Agents and the Origins of Atherosclerotic Coronary Artery Disease

**DOI:** 10.3389/fcvm.2016.00030

**Published:** 2016-09-12

**Authors:** James S. Lawson

**Affiliations:** ^1^School of Biotechnology and Biomolecular Sciences, University of New South Wales, Sydney, NSW, Australia

**Keywords:** atherosclerosis, coronary disease, infectious pathogens, diet and nutrition, coronary atheroma, trends

## Abstract

Although deaths due to atherosclerotic coronary artery disease (ACAD) have fallen dramatically during the past 50 years, ACAD remains as the leading cause of death in all continents, except Africa, where deaths due to infections are still dominant. Although food and nutrition have a proven role in atherosclerosis, the underlying causes of ACAD remain unknown. This is despite a century of intensive research dominated by investigations into the saturated fat hypothesis. In this review, it is hypothesized that the rise and fall in ACAD during the past 100 years is primarily due to the parallel rise and fall in the prevalence of coronary atheroma, the underlying disease. It is further hypothesized that infectious pathogens initiate atherosclerosis mainly during infancy and childhood. It is speculated that widespread use of antibiotics and vaccines against bacterial and viral infections may be the reason for the dramatic fall in coronary atheroma and ACAD during the past 50 years. The relevant evidence and a working hypothesis are included in this review.

*Chlamydia pneumoniae*, cytomegalovirus, hepatitis C, Epstein Barr virus, human papilloma virus, human immunodeficiency virus, enterobacteriaceae, and other infectious pathogens may initiate atherosclerotic vascular diseases.

## Hypothesis

The main risk factors for atherosclerotic coronary artery disease (ACAD) are genetics, abnormal blood lipids, hypertension, diabetes, tobacco smoking, and glucose intolerance. In addition to these well-documented risk factors, it is hypothesized that (i) ACAD is initiated by multiple infectious pathogens, (ii) these pathogens have adverse influences on the endothelial lining and smooth muscle cells of blood vessels, which lead to inflammation of the vascular system, (iii) some pathogens influence lipid metabolism, (iv) several pathogens may live in the vascular system for decades and cause atheroma, (v) there is an influence of food on these pathogens and the formation of atheroma, and (vi) total food consumption has an additional independent influence on ACAD.


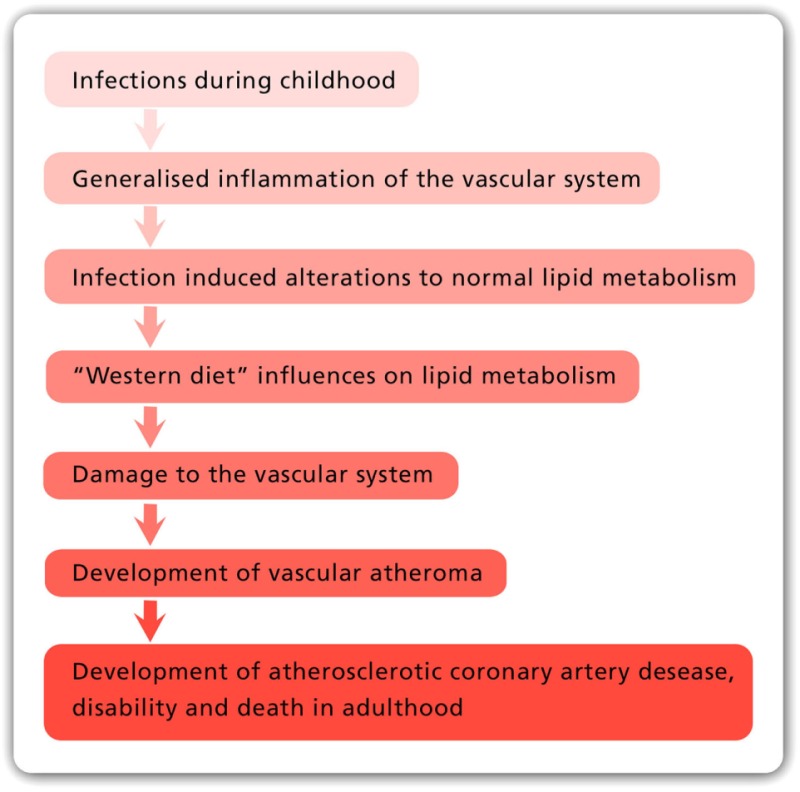


## Background

Atheroma or atherosclerosis is a chronic inflammatory disease of the arterial wall (1). Atherosclerosis in arterial walls is characterized by the invasion and accumulation of white blood cells, which transform into macrophages. Macrophages ingest lipids becoming “foam” cells. Concurrently, there is proliferation of vascular smooth muscle cells, resulting in the creation of fibrofatty plaques. These changes result in thickening of the walls of arteries. The early signs of atherosclerosis are fatty streaks lining the arteries, which years later, develop into plaques. These plaques may rupture and induce thrombus formation in the lumen of an artery, which may lead to thromboembolism or arterial occlusion with consequent coronary ischemia and death (1).

There is sound evidence that atherosclerosis is initiated in infancy and childhood and is associated with childhood infections that develop later age atheroma (2–5). Autopsy based data have shown that ACAD can begin in the perinatal period and early childhood and can be well established by the teenage years (2, 3, 6, 7). Several of these studies are based on retrospective observations of the same subjects over a 30-year period and indicate that there may be an over two times increased risk of cardiovascular disease in adulthood following a serious (sufficient to cause hospitalization) childhood infection (4, 5). The identification of specific pathogens was not made in these studies. Many Western children may have early and even advanced atheroma at ages 12–14 years (2, 3, 7, 8). Autopsy-based studies of young US white and black subjects, killed between 1987 and 1994 because of accidental trauma or homicide, have demonstrated that over half of the right coronary artery of the youngest age group (15–19 years) had atheromatous lesions (8). This is similar to the coronary atheroma lesions observed in young (average age 22–27 years) German (1914) killed in World War I and US soldiers killed in the Korean (1951–1953), Vietnam (1965–1975), and the Afghanistan and Iraq wars (2001–2011) (9–12).

**Figure 1:**
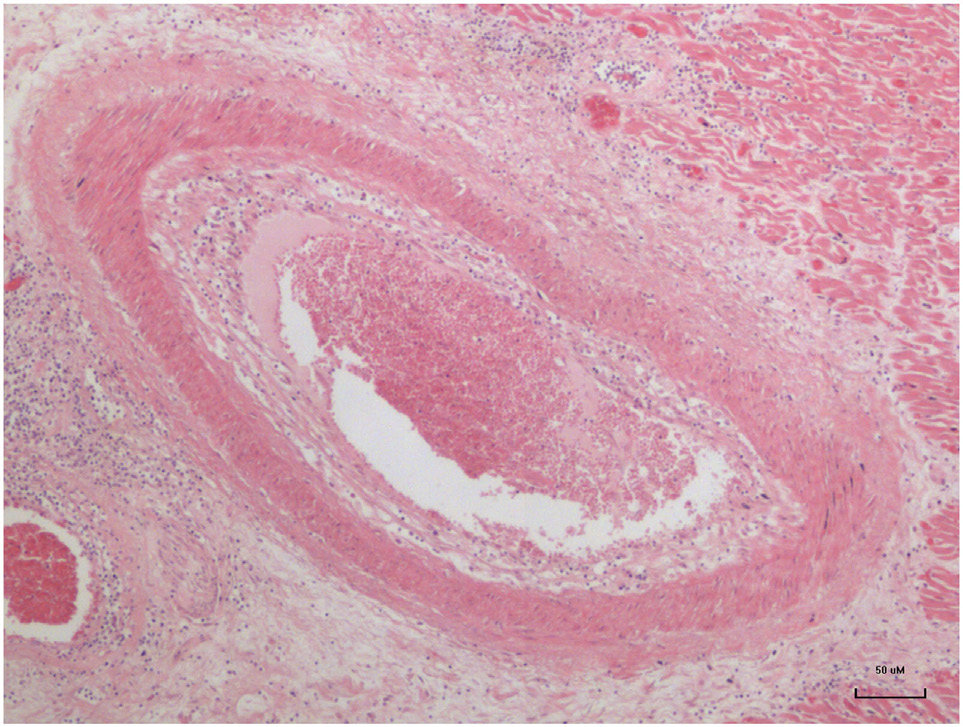
**Advanced atheroma in the coronary artery of a 17–year-old girl (13)**.

Advanced atheroma of a coronary artery in a 17-year-old girl is shown in Figure 1.

### Trends in the Prevalence of Atherosclerosis and the Search for the Underlying Causes of Atherosclerosis

**Figure 2:**
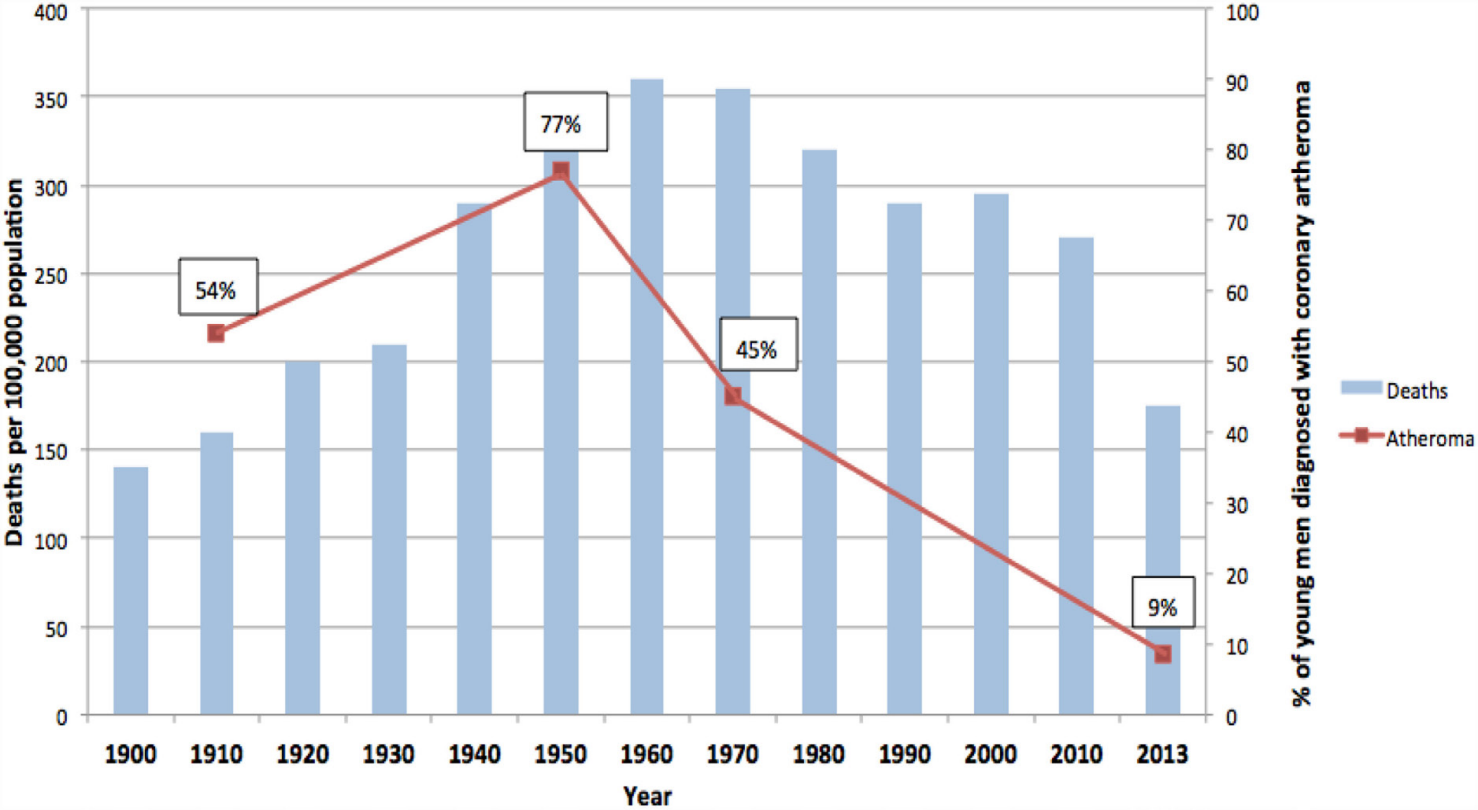
**Deaths due to coronary artery disease USA (blue columns). Coronary atheroma prevalence (red line). 100-year trends (9–12, 15)**.

The rise and fall in the number of deaths due to ACAD over the past 100 years in the US is typical of other Western countries including the United Kingdom, Germany, and Australia (14). Data from the US are of special value as they are available for over 100 years. As shown in Figure 2, deaths in the US due to ACAD rose from approximately 140 per 100,000 population in 1900 to a peak of 355 in 1960 followed by a remarkable decline to 170 in 2010 (15). These data are based on variations of the diagnosis of heart disease and must be regarded as trends rather than precise figures. However, the historical data are broadly in line with more recent data based on specific diagnoses of coronary artery disease and age-adjusted annual death rates per 100,000 of the total US population (16). These later data indicated a 76% decline during the past 50 years (16).

In addition to this pattern of deaths due to coronary artery disease, there has been an equally dramatic fall in the prevalence of atheroma of the coronary arteries. This is also shown in Figure 2. The data in Figure 2 are based on the direct observation of coronary arteries during autopsies conducted on soldiers killed in wars over a 100-year period. These autopsies clearly demonstrate coronary atheroma in physically fit young men (average age 22–27 years) from Germany (First World War – 1915) and the US (Korea 1950–1953, Vietnam 1964–1975, Iraq and Afghanistan 2001–2011) (9–12). These data are shown in more detail in Table 1.

**Table 1:** **Prevalence of atheromatous coronary artery disease in young Western soldiers (over 98% males) killed in World War I, Korea, Vietnam, Iraq, and Afghanistan (9–12)**.

Age groups deceased soldiers	1915 World war I *n* = 171 (mostly German soldiers) (i)	1950–1953 Korea *n* = 300 (US soldiers) (ii)	1964–1975 Vietnam *n* = 105 (US soldiers) (iii)	2001–2011 Iraq and Afghanistan *n* = 3,832 (US soldiers) (iv)
<25 years	22/50 (44%)	na	na	135/2,047 (7%)
25–29 years	23/47 (49%)	na	na	103/931 (11%)
30–39 years	21/35 (60%)	na	na	154/697 (22%)
40+ years	26/39 (67%)	na	na	72/157 (46%)
Average age	29 years	22 years	22 years	26 years
Atheroma all ages (%)	92/171 (54%)	232/300 (77%)	47/105 (45%)	464/3,832 (12% aorta + coronary; 9% coronary)

*The age specific data, based on autopsies of soldiers killed in World War I and in the Afghanistan and Iraq wars, are of particular interest because of the clear demonstration that the prevalence of coronary atheroma increases markedly with age*.

*na, not available*.

The data based on each war period are not strictly comparable due to different diagnostic criteria and autopsies conducted by different pathologists. Therefore, the validity of the data, based on autopsies of soldiers over a 100-year period, needs to be considered. First, the diagnosis of coronary atheroma by gross observation and light microscopy is not difficult and is reasonably consistent. Second, the prevalence of coronary atheroma based on autopsies in male soldiers in 1950–1953 was broadly similar to that observed in military pilots in 1980–1982 (17) and to those made on US civilians between 1950 and 1994 (18). Among the military pilots, Booze and Staggs observed a fall of severe coronary atherosclerosis from 5 to 2.5% between 1975–77 and 1980–82 (17). Third, based on uniform autopsy techniques in studies conducted in New Orleans, the extent and prevalence of atherosclerotic lesions in the coronary arteries and aortas of deceased 25- to 44-year-old white men significantly decreased between 1960–64 and 1969–78 (19). These declines were not observed among same aged black men. Fourth, and also based on a uniform system of autopsy techniques, the prevalence of coronary atheroma among men and women below the age of 60 years decreased from 38 to 27% during the period 1979–1994 in Olmsted County, MN, USA (20). There was a further decline in coronary atheroma of approximately 20% among the same population between 1995 and 2012 (15). The decline in prevalence of coronary atheroma in Olmsted County was greater among young as compared to older citizens. The rate of decline in coronary atheroma has slowed in recent years (15). Because these data are based on a wide range of age groups, they are not directly comparable to the age-specific data based on US soldiers. However, the decline in coronary atheroma demonstrated by the New Orleans and Olmsted autopsy-based data parallels and adds validity to the US soldier-based data (15, 19). Overall, the data from different sources are consistent, particularly with respect to the decline in prevalence of coronary atheroma among young men. It is concluded that the decline in coronary atheroma is real and not due to different methods of assessing autopsy findings.

The rise and fall in the prevalence of coronary atheroma closely parallels the prevalence of deaths due to coronary artery disease in the US during the same periods of time (see Figure 2) (15). This decline in deaths due to ACAD is in part due to a reduction in risk factors and improvements in surgical and medical treatments. However, as hypothesized in this review, it is likely that the decline in the underlying disease, namely atherosclerosis, has been the dominant factor. The fall in deaths due to coronary artery disease by approximately two-thirds during the past 50 years has occurred in all Western countries (21). The epidemiological trends of deaths due to coronary artery disease are different in Eastern European, South American, and Asian populations (21). The pattern of a dramatic rise and fall in deaths is typical of an infectious-based disease.

It should be noted that (i) different methodologies and assessments by pathologists over a 100-year period apply to the atheroma autopsy-based studies (red line) and (ii) different criteria apply to the deaths attributed to coronary artery disease (blue columns). Accordingly, these data indicate trends and not precise measurements.

### Infectious Pathogens

The classic risk factors for atherosclerosis are well established (22, 23). These risk factors include excess body weight, dyslipidemia, hypertension, tobacco smoking, genetics, diabetes, and glucose intolerance. An important feature of atherosclerosis is the marked increase in prevalence associated with increased age. This indicates an ongoing chronic process.

In addition to these risk factors is the likely role of infectious pathogens. The first experimental evidence that infectious pathogens could cause atherosclerosis in blood vessels was developed by Fabricant et al. in 1978 (24). They demonstrated that Marek’s herpes virus could cause atherosclerosis in chickens. Infection with this herpes virus was required to induce atherosclerosis whether or not the chickens were fed a high or low cholesterol diet. They also demonstrated that infections with these herpes viruses altered intracellular lipid metabolism (25). Importantly, this infectious phenomenon could be prevented by vaccination (26). These observations of atherosclerosis in chickens closely resemble atherosclerosis in humans and are a key to the hypothesis that infectious pathogens may have a causal role in vascular atherosclerosis in humans (26).

A number of herpes viruses have been identified in atherosclerotic coronary arteries and normal vascular structures in humans. These herpes viruses include Epstein–Barr virus (EBV) (human herpes virus 4), Cytomegalovirus (CMV) (human herpes virus 5), and herpes simplex virus (HSV) (human herpes virus 1 and 2) (13, 27, 28). Infections with EBV and other herpes viruses are associated with atherogenic lipid changes (29–32).

Multiple additional pathogens in both normal and atherosclerotic blood vessels have been identified (33, 34). The pathogens identified in normal blood vessels by a species-specific approach include *Chlamydia pneumoniae* (Cp), *Helicobacter pylori, Mycoplasma pneumoniae*, CMV, hepatitis C, HSV, EBV, human papilloma virus (HPV), and human immunodeficiency virus (HIV) (13, 27, 35, 36). There is an extensive list of additional pathogens, which have been identified in atherosclerotic plaques by massive computerized gene sequencing (34). These include the pathogen families, such as porphyromonadaceae, bacteroidaceae, micrococcaceae, streptococcaceae plus viruses such as EBV and HPVs. The importance of these observations is (i) they demonstrate that the long held view that blood vessels were sterile is incorrect and (ii) that pathogens in blood vessels are not necessarily parasites, which infect atheromatous plaques.

### Epidemiological Evidence for a Role of Infectious Pathogens in Atherosclerosis

There is epidemiological evidence, which is supportive of a role by several specific pathogens in atherosclerosis.

#### Periodontal *Porphyromonas gingivalis*

Pathogens associated with periodontal infections have been extensively studied. A higher periodontal pathogen load with *Porphyromonas gingivalis* has been identified in patients with atherosclerotic vascular disease as compared to controls (33, 37).

#### Human Papilloma Virus

Women with HPV genital infections have up to threefold increased risk of atherosclerotic vascular disease (38), and high risk for cancer HPVs have been identified in atheromatous plaques and in coronary artery smooth muscle cells (13). There is experimental evidence that human aortic smooth muscle cells proliferate when exposed to HPVs (39).

#### *Chlamydia* *pneumoniae*

There are many studies, which confirm the presence of Cp in atheroma plaques (40). In a large study of Japanese men and women, high levels of serum antibodies to Cp were associated with a doubling of the prevalence of ACAD (41). These findings, suggesting a role for Cp in atherosclerosis, have been supported by recent experiments, which show that a single infection of Cp is sufficient to exacerbate atherosclerosis in mice (42).

#### Enterobacteriaceae

There is a possible link between the identification of enterobacteriaceae in vascular atherosclerotic lesions and serious childhood infections with later adult ACAD (4, 5, 43). This is because many serious childhood infections are caused by enterobacteriaceae in the renal tract.

### Infections and Chronicity

It is possible that chronic infections may have a role in the marked increase in the prevalence of ACAD between the ages of 20 and 40 years. This increase in ACAD associated with age was clearly demonstrated by the study of atherosclerosis in US soldiers killed in the Iraq and Afghanistan wars (12). While the reasons are not known, Cp has the capacity to cause inflammation over decades of time. Similarly, high risk for cancer HPVs may cause persistent inflammation and a tripling of the risk of ACAD in women (13, 38). CMV and EBV also have the capacity to persist over decades (35, 44).

### Microbiota

Microbiota refers to the hundreds of different bacteria and viruses that are located throughout the body. The largest and most diverse microbiota location is the gut. While the evidence is not definitive that the gut or other microbiota influence the development of atherosclerosis, there is substantial evidence in support of this notion (45, 46). There are different microbiotas in different locations, including the genital tract, blood, and even atherosclerotic plaques (46). On the other hand, atherosclerotic plaques, the gut, and periodontal locations can have similar microbiota in the same patients (46). This microbiota can be dominated by *Escherichia coli* (46). This suggests that there may be similar biological influences in these very different locations. The composition of the gut microbiota is influenced by diet and infectious pathogens and also by antibiotics (47, 48).

Of particular interest is the relationship between viruses and bacteria in the gut (49). Several viruses, including noroviruses (the cause of 85% of gastroenteritis cases), mouse mammary tumor virus (the cause of breast cancer in mice and other mammals probably including humans), poliomyelitis virus, reovirus (the cause of respiratory and gastrointestinal infections), each require the co-location of bacteria in the gut to allow these viruses to enter cells (49). The special interest is because in experiments in which these viruses were exposed to antibiotics that inhibited gut bacteria, these viruses could not enter the target cells (49). This is relevant to the hypothesis that antibiotics may be one reason for the decline in the prevalence of atherosclerosis.

### Food

For over 50 years, food consumption patterns have been the dominant causal hypothesis for vascular atheroma. Overall, the epidemiological studies on humans and the experimental studies on animals offer consistent observations that food consumption patterns are associated with coronary artery disease. The caution expressed decades ago by Marmot and Syme (50) and Truett et al. (51), that ACAD had more complicated origins than simply diet, was especially farsighted because at the time of their writing, it was not known that pathogenic infections could cause changes in lipid metabolism.

There has been a dominance of the saturated fat and coronary heart disease hypothesis. This is mainly due to the studies and promotion by Keys and his colleagues (52). This hypothesis was based on Keys’ famous Seven Country Study, which demonstrated that coronary heart disease was most common in Western dairy and red-meat eating countries and was associated with high blood lipid levels (52). During the same period, prospective studies were conducted in the town of Framingham in the US (53). These studies showed that high blood pressure, high blood cholesterol levels, obesity, and tobacco smoking were associated with increased risk of coronary artery disease (53). Despite the clear statements from the Framingham research group that evidence was not available upon which to base action, for the next 50 years, the views of the Ancel Keys research group became the basis of formal dietary guidelines in most Western countries. These guidelines included dogmatic advice for citizens of Western countries to reduce consumption of saturated fats, including most dairy foods. The assumption by the Ancel Keys group that a reduction in dietary saturated fats on a population basis would lead to a reduction in serum cholesterol is flawed. This is because cholesterol synthesis is regulated by a system of enzymes, which maintain the body’s cholesterol levels within a narrow range. When saturated fat intake increases, endogenous cholesterol production decreases. When the intake of saturated fat is decreased, endogenous production increases to maintain serum cholesterol levels relatively constant (54). It should be noted that while it is possible to lower serum cholesterol in individuals by diet, the required diets are so severe that they are not sustainable.

The findings of these early studies, which demonstrated the influence of food consumption patterns on atherosclerosis, have been repeatedly confirmed. However, many studies sought to emphasize a potential harmful role of saturated fats. This emphasis has been shown to be misleading, and there is now a substantial body of evidence available that demonstrates the limited role of saturated fats in atherosclerosis (55–58). The recent Cochrane Collaboration (59) review of the evidence relevant to this issue concluded “…cutting down on saturated fat led to a 17% reduction in the risk of cardiovascular disease (including heart disease and strokes) but no effect on the risk of dying.”

On the other hand, there is compelling epidemiological evidence that total food consumption, as distinct from specific components of food, is associated with the prevalence of ACAD. In both low and high risk for ACAD countries, an increase in the body mass index is positively associated with increased incidence of ACAD (22). Of particular interest is the recent 40-year prospective nationwide study of approximately 2.5 million Israeli adolescents, which demonstrated that overweight Israeli teenagers were almost twice as likely and obese teenagers were up to five times as likely to have a fatal heart attack or stroke during middle age (60).

For over five decades, there has been a consistent association between total per capita food consumption and ACAD in both low and high risk for ACAD countries. This is shown in Table 2. In 1961, the daily apparent consumption of calories in South Korea was 2,141 as compared to over 3,000 in US, which positively correlates to the 1993 death rates due to coronary artery disease of 13.5 in South Korea as compared to 107.5 per 100,000 total population in the US (61, 62). As also shown in Table 2, these trends have persisted to the present time with a doubling of deaths associated with coronary artery disease in Korea, associated with a marked increase in food consumption. By way of contrast, death rates due to coronary artery disease have halved in Australia, the United Kingdom, and the US, despite only modest increases in food consumption.

**Table 2:** **Asian (South Korea, Japan) and Western (Australia, United Kingdom, US) apparent daily per capita food consumption in kilocalories 1954/1961 and 2006/2008 and age-adjusted death rates per 100,000 population due to coronary artery disease 1993 and 2014 (61, 62)**.

	Daily kilocalories 1954/1961	Daily kilocalories 2006/2008	Deaths coronary artery disease 1993	Deaths coronary artery disease 2014
South Korea	2,141	3,040	13.5	26.4
Japan	2,070	2,800	21.5	30.4
Australia	3,230	3,220	112.6	54.9
United Kingdom	2,570	3,450	118.9	60.1
US	3,150	3,800	107.5	78.0

The prevalence of ACAD based on autopsy findings among Japanese living in Hawaii and California is up to 3.5 times more frequent and more severe than Japanese living in Japan (63, 64). Serum cholesterol was significantly higher in the Japanese Hawaiians and Californians. However, as Marmot observed, when Japanese men ate equivalent diets in the three places, the Japanese Americans still had higher serum cholesterols than the Japanese in Japan (50). The implication being that serum cholesterol is due to factors other than fat consumption.

The patterns of food consumption in the US offer important support for the view that the components of diets, such as saturated fats and cholesterol, have little association with the prevalence of atheromatous coronary artery disease. These patterns are shown in Table S1 in Supplementary Material. In the US, during the past 50 years, food energy has increased by 25%, protein consumption by 32%, and total fats by 38%. Consumption of cholesterol and added sugars has been stable. The overall patterns of food consumption shown in Table S1 in Supplementary Material parallel the high prevalence of obesity in the US. During this same period, the consumption of saturated fats increased by approximately 10% (Table S1 in Supplementary Material). These US food consumption trends are opposite to what would have been expected if the saturated fat and atheroma hypothesis had been true.

## Mechanisms

### Infectious Pathogens

There are several excellent reviews of the role of infectious pathogens and atherosclerosis by Epstein et al., Campbell and Rosenfeld, Feingold and Grunfeld, and Clifford and Hoffman (65–68). These reviews, plus additional material, have been used to explore the underlying mechanisms of infections and atherosclerosis as outlined below.

Multiple infectious pathogens contribute to acute and chronic inflammation of the vascular system (66). Although the evidence is not entirely consistent, infections of vascular wall cells lead to inflammation and atherosclerosis. The specific mechanisms differ between various pathogens. Both CMV and Cp appear to induce proliferation of vascular smooth muscle cells by inhibition of the tumor suppression gene p53 (69, 70). CMV, Cp, and HSV can each have an atherogenic effect, including smooth muscle proliferation, increased expression of cytokines, chemokines, and an increased uptake of low density lipoprotein (71).

There also appear to be infectious pathogen-associated autoimmune mechanisms associated with atherosclerosis. These mechanisms are complex and involve the increased expression of heat shock proteins (HSPs) in response to stress, such as infections. In some circumstances, these proteins may appear as “foreign” and engender an autoimmune response. Many studies support the validity of this mechanism, including the encoding by all bacteria of HSPs (65). In addition, serum antibodies to HSPs are associated with carotid artery thickening, and serum antibodies to HSPs of *E. coli* and Cp are cytotoxic to vascular endothelial cells (65). It is also possible that viruses can evoke an HSP autoimmune response (64). An additional immune-based mechanism by which infectious pathogens may contribute to atherosclerosis is *via* toll-like receptors (TLRs). Pathogens can increase expression of TLRs and engender a chronic inflammatory response (65).

Infections and inflammation can have complex influences on lipids and lipoproteins (67). The most common influences are decreases in serum high density lipids and increases in triglycerides. The greater the severity of inflammation, the greater the abnormalities in lipids and inflammation. The underlying mechanisms are not clear.

Infections leading to vascular inflammation appear to have the capacity to influence risk factors associated with atherosclerosis.

### Infections and Diet

There may be a connection between food consumption patterns, infection, and atherosclerosis. However, the evidence is very limited. Studies based on experimental animals suggest that the influence of infectious pathogens may differ according to the specific pathogen, the experimental animal, and type and quantity of food. Fabricant et al. demonstrated that Marek’s herpes virus leads to atherosclerosis in both hypercholesterolemic and normocholesterolemic chickens (24). Similarly, *P. gingivalis* bacteremia has been shown to induce coronary and aortic atherosclerosis in both normocholesterolemic and hypercholesterolemic pigs (72). Turkay et al. demonstrated that in experimental rats, a combination of a high cholesterol diet and infection with *Pseudomonas aeruginosa* led to significantly greater arterial wall thickness than either infection or cholesterol diet administered alone (73). There are several experimental animal-based studies, which suggest that Cp is a risk factor for atherosclerosis in conjunction with hyperlipidemia (66).

There is limited relevant evidence in humans. Amazon forage horticultural people have high loads of infection, but low adiposity and virtually no arterial degeneration and ACAD (74, 75). These observations suggest that infections alone may not cause atheroma in humans, and that “excess” food and nutrition plus infectious pathogens are also required. A precise definition of “excess” nutrition is not possible, but the average daily consumption of food in Western populations is over twice that of Amazon villagers (75).

### Infectious Pathogens, Inflammation, and Lipid Metabolism

Because of the important role of lipids in vascular atherosclerosis, the role of specific infectious pathogens in the disruption and influence on lipid metabolism is relevant to the search for the underlying causes of atheroma (67). In Table 3, potential pathogens are listed together with their known influences on human and animal lipid metabolism.

**Table 3:** **Influence of pathogens on human and experimental animal lipid metabolism**.

Pathogen	Abnormal lipid metabolism humans	Abnormal lipid metabolism experimental animals
*Chlamydia pneumonaie*	+	+
*Porphyromonas gingivalis*	+	na
Epstein–Barr virus	+	na
Hepatitis C	+	na
Cytomegalovirus	+	na
*Helicobacter pylori*	+	na
Human papilloma virus	+	na
*Enterobacter coli*	na	na
Marek’s herpes virus	na	+
Herpes viruses	+	na
Human immunodeficiency virus	+	na

*+, disruption or abnormal lipid metabolism, na, data not available*.

#### Human Immunodeficiency Virus

The influence of HIV to increase the risk of ACAD has been studied in detail. Chronic HIV infections are associated with abnormal lipid metabolism, which in turn is associated with increased ACAD (36, 76).

#### *Chlamydia* *pneumonaie*

*Chlamydia pneumonaie* disrupts lipid metabolism in human umbilical vein endothelial cells and is associated with increased levels of total cholesterol in these cells (77). Cp-infected carotid artery macrophage foam cells contain abnormally high low density lipoproteins (78). Cp infections trigger the formation of lipid-laden foamy macrophages, as seen in atheromatous plaques (79). Cp-infected mice show significantly increased serum cholesterol and triglyceride levels compared to controls (80). Cp infections accelerate the development of atherosclerotic lesions in experimental mice fed with high-fat diets (81).

#### *Porphyromonas* *gingivalis*

Chronic oral infection with *P. gingivalis* accelerates atheroma formation by altering lipid metabolism (82). In addition, *P. gingivalis* increases lipid accumulation in foam cells (83).

#### Epstein–Barr Virus

Acute infection with EBV is associated with atherogenic lipid changes (30). EBV may also be involved in the inflammatory and autoimmune processes associated with atherosclerosis (84).

Hepatitis C alters host cell lipid metabolism (85).

Human CMV infections influence host cell sphingolipids and lipid metabolism (86, 87).

*Helicobacter pylori* influences serum lipids and appears to be associated with ACAD (88).

Herpes viruses influence arterial accumulation of cholesterol (29).

Human papilloma virus influences intracellular lipids (89).

Human immunodeficiency virus influences lipid metabolism and increases cardiovascular disease (90).

## Reasons for the Dramatic Decline in Atherosclerosis and the Fall in Deaths Due to Atherosclerotic Coronary Artery Disease

Determining the etiology of atherosclerosis is a complex task. Therefore, it is helpful to consider Karl Popper’s concept of falsifiability or refutability of the hypotheses outlined in this review (91). These hypotheses will be nullified or falsified if they are shown to be invalid or false.

The decline in the prevalence of deaths due to ACAD during the past 50 years is extraordinary. A range of reasons for this dramatic decline have been offered (92). These include reductions in total serum cholesterol (24%), tobacco smoking (12%), blood pressure (20%), and physical activity (5%) plus treatments associated with revascularization (15%), heart failure (9%), and other therapies (12%) (92). These explanations are well documented and appear to be valid. Importantly, none of these risk factors are relevant in infants, children, and young adults, among some of whom atheroma can be well established before the age of 22 years (12, 17, 18). There must be additional explanations.

### Food

While total excess energy consumption is associated with the formation of vascular atheroma, there is limited evidence to support the hypothesis that saturated or other dietary components are major contributors. The substantial increases in consumption by US citizens during the past 50 years, of all types of fats, proteins, and to some extent sugars, is the opposite of what would be expected with such a dramatic decline in vascular atheroma. This is in accord with Popper’s concept of falsification.

### Tobacco

There has been a substantial decline in tobacco smoking by US citizens during the past 50 years. Forty-two percent of US adults smoked tobacco in 1965, and this had fallen to 19% in 2011 (93). It is likely that this has contributed to the decline in deaths due to coronary artery disease due to atherosclerosis, thrombosis, and coronary occlusion (94). However, this decline in tobacco smoking is unlikely to be a major factor in the substantial decline in atheroma in young soldiers (because of their extremely young average age) (12). Again, this is in accord with Popper’s concept of falsification.

### Hypertension

There is strong evidence that population-wide control of hypertension has resulted in a decline in deaths due to coronary artery disease (92). High blood pressure was associated with coronary atheroma in 43% of the US soldiers (although the numbers of soldiers with hypertension was small and unlikely to be present in the young soldiers and is again in accord with Popper’s concept of falsification) (12). The prevalence of hypertension among US adults has fallen and then risen during the past 50 years (95). It is arguable that the control of hypertension has contributed to the decline in atheroma, but given the rise in the prevalence of hypertension in the US over the past 30 years, this is doubtful.

### Statins

Statins have been widely available for the past 25 years. Statins can lower blood lipids by up to 50% and significantly reduce atherosclerotic plaques and have been shown to significantly reduce ACAD (96). The use of statins has illustrated the important associations between blood lipids and cardiovascular disease (96). However, the introduction of statins is relatively recent and well after the decline in coronary atheroma and ACAD. Importantly, statins are not prescribed for young adults and cannot have had a role in the reduction of coronary atheroma in this age group. Again, this is in accord with Popper’s concept of falsification.

### Antibiotics and Vaccines

Antibiotics and viral vaccines against a range of infectious diseases began to be used on a population-wide basis in the post World War II years. There is no direct evidence that antibiotics and viral vaccines have had an influence on atherosclerosis in humans, but it a plausible explanation as both have been shown to reduce atheroma in experimental animals (26, 97, 98). A majority of children have been exposed to antibiotics following their widespread use since the 1950s. Many types of bacteria that have been identified in human blood vessels are sensitive to antibiotics, although bacteria, such as Cp, are less sensitive but can be controlled by broad spectrum antibiotics.

There is a major impact on the commensural gut bacteria (the gut microbiota) by broad spectrum antibiotics (99, 100). Different antibiotics have different influences on specific components of the gut microbiota (99). The influence of antibiotics is well illustrated by the impact of maternal intrapartum antibiotics on gut microbiota, which persist during the first year of life (48). In experimental animals, antibiotics have been shown to alter bacterial composition and short-chain fatty acids in the cecum (100). Experiments on mice have demonstrated that antibiotics change the gut microbiota, which in turn lead to improvements in glucose tolerance, reduced plasma lipopolysaccharides, and altered hepatic and intestinal genes involved in inflammation (98). While the evidence is very limited, it is possible that antibiotics have an influence on gut microbiota, which in turn may have an influence on blood lipids and consequently on atherosclerosis.

It is also possible that vaccines against viral infections may prevent atheromatous processes. This hypothesis is based on the use of vaccines against Marek’s herpes virus, which was shown to prevent herpes-induced atheroma in chickens by Fabricant and Fabricant (26).

As outlined above, several viruses, including noroviruses, mouse mammary tumor virus, poliomyelitis virus, and reovirus, each require the co-location of bacteria in the gut to allow these viruses to enter cells (49). There is experimental evidence that when these viruses were exposed to antibiotics that inhibited gut bacteria, these viruses could not enter the target cells (49). This is relevant to the hypothesis that antibiotics and vaccines may independently be the reasons for the decline in the prevalence of atherosclerosis.

## Conclusion

There has been a substantial (70% among young people) decline in atherosclerosis of the coronary arteries during the past 50 years. There has been an equivalent decline in deaths due to ACAD in Western countries. Despite these declines, ACAD remains as the most common cause of death in all continents except Africa. The reasons for these declines are not clear. A plausible, but unproven, explanation is the widespread introduction of antibiotics and vaccines in the post second world war years.

While food and nutrition are important influences on atherosclerosis, there is mounting evidence that infectious pathogens may initiate atherosclerosis. These influences commence in infancy and childhood, and atherosclerosis can be well established in some children as young as 12 years of age and in otherwise fit young adults. Many pathogens influence lipid metabolism, for example Marek’s herpes virus, can cause atherosclerosis in experimental chickens in the absence of atherogenic diets. Multiple pathogens can infect both the normal and atherosclerotic vascular system, including the aorta and coronary arteries, having adverse influences on the endothelial lining and smooth muscle cells of blood vessel walls leading to inflammation.

There is evidence, which is not conclusive, that the herpes viruses, Cp, *P. gingivalis*, and *Enterobacter coli* may be the most influential pathogens associated with atherosclerosis.

The search for the causes of ACAD has been dominated by research into the influence of food and nutrition. It is timely to redirect research efforts.

## Recognition

The young soldiers killed in World War 1, Korea, Vietnam, Iraq, and Afghanistan are not just data and statistics. Necessarily, in this review, they are anonymous but not unknown. Sadly, they are sons, daughters, brothers, and sisters, and some are husbands and wives.

## Author Contributions

The author confirms being the sole contributor of this work and approved it for publication.

## Conflict of Interest Statement

The author declares that the research was conducted in the absence of any commercial or financial relationships that could be construed as a potential conflict of interest.
